# Efficacy of the Granulocyte Colony-Stimulating Factor in Sepsis-Associated Immunosuppression: An Open-Label Randomized Controlled Trial

**DOI:** 10.7759/cureus.104055

**Published:** 2026-02-22

**Authors:** K Keerthi Reddy, Sunil Kumar Rao, Anil Kumar Saroj, Chandradeep Srivastava, Tej Bali Singh, Kamlesh M Palandurkar

**Affiliations:** 1 Department of Pediatrics, Institute of Medical Sciences, Banaras Hindu University, Varanasi, IND; 2 Center for Biostatistics, Institute of Medical Sciences, Banaras Hindu University, Varanasi, IND; 3 Department of Biochemistry, Institute of Medical Sciences, Banaras Hindu University, Varanasi, IND

**Keywords:** granulocyte colony-stimulating factor, hospital acquired infection, pediatric sequential organ failure assessment score, sepsis-associated immunosuppression, tumor necrosis factor-alpha

## Abstract

Objective: To determine the efficacy of filgrastim (G-CSF) in reducing the mortality in children with multi-organ failure syndrome (MOFS) persisting for three consecutive days.

Methods: Children aged 1 month to 18 years with two or more organ failures persisting for three days according to Goldstein’s criterion were included and randomized to receive either standard of care and filgrastim (G-CSF) at a dose of 4 mcg/kg/day subcutaneously for seven days or standard of care at a 1:1 ratio. The stored blood samples were estimated for TNF-α, A Disintegrin and Metalloproteinase Motifs 13 (ADAMTS13), and soluble Fas ligand (FasL) at the end of the study to confirm the biomarker-based inflammatory phenotypes of sepsis-induced MOFS. Outcomes were 28-day mortality and differences in tumor necrosis factor-alpha (TNF-α) levels, hospital-acquired infection (HAI), and pediatric sequential organ failure assessment score (pSOFA) at seven days of randomization.

Results: Of 78 children, 25 (32%), 50 (64.1%), and 3 (3.8%) were discharged, died, and left against medical advice (LAMA), respectively. The two groups were similar except for a higher TLC (14100 [11400-16270]) vs. (17560 [13900-22100]; p=0.02) and male preponderance (18/39 vs. 27/39; p=0.03) in the control group. The intervention group received 2 (2-3) median (IQR) doses of filgrastim (G-CSF) for a 3 (2-3) median (IQR) duration of days. No significant difference was observed between the groups regarding 28-day mortality (26/39 vs. 27/39; 95% CI, p (0.71-1.31, p=0.81), HAI (31/62 vs. 21/53; p=0.27). The pSOFA scores and TNF-α levels at seven days were 8 (6-12) vs. 9 (8-11) (p=0.12) and 81.6 (6.9-237.2) vs. 99.6 (16.2-404.2) (p=0.29), respectively. Subgroup analysis revealed a similar occurrence of mortality in immunoparalysis-associated multi-organ failure (IPMOF) (16/26 vs. 14/21); 95% CI, p (0.60-1.42, p=0.71) at 28 days, and 2 median dose of filgrastim (G-CSF) for 3 median day did not significantly change the TNF-α levels within the intervention group at day seven (56 [32-118] vs. 19 [6.9-118], p=0.77) as compared to day 0. We did not observe any life-threatening/significant sudden deterioration/anaphylaxis after use of filgrastim (G-CSF).

Conclusions: Filgrastim (G-CSF) use in immunocompetent children with sepsis-induced MOFS is safe, and a 2-dose of filgrastim (G-CSF) for three days has poor efficacy in the reduction of mortality and did not show significant change in frequency of HAI, TNF-α levels, and pSOFA scores.

## Introduction

Despite advancements in antimicrobial treatments and supportive care, sepsis remains a leading cause of death globally. In 2017, an estimated 25 million children experienced sepsis worldwide, leading to more than 3 million deaths [1]. The hyperinflammation, immunosuppression, or mixture of both occurs concurrently in the early stages of sepsis. The pathobiological mechanisms and clinical features of sepsis present differently in each patient and exhibit clusters or phenotypes that may respond differently to therapies. The three inflammatory phenotypes of multi-organ failure syndrome (MOFS) were identified in septic children; these are immunoparalysis-associated multi-organ failure (IPMOF), thrombocytopenia-associated multi-organ failure (TMOF), and sequential multi-organ failure (SMOF) [2,3]. The clinical criteria of IPMOF, TAMOF, and SMOF were proposed by Carcillo et al., and levels of TNF-α (<200 pg/ml), ADAMTS13 activity (<57% of control), and Fas L (>200 pg/ml) confirmed the inflammatory phenotypes. The prevalence of IPMOF varies from 21.1% to 34% in developed countries, whereas it is 28.6% in an Indian study [3-6]. IPMOF is a state of suppression of innate immunity by heterogeneous hyperinflammatory responses secondary to sepsis, which reduces the expression of human leukocyte antigen-DR (HLA-DR) on monocytes and the release of pro-inflammatory cytokine (TNF-α) by monocytes. As a consequence, septic children with IPMOF are unable to eliminate primary infection and are susceptible to nosocomial infection, persistence of MOFS, and adverse outcome [4,6]. Granulocyte-colony stimulating factor (G-CSF) or granulocyte-macrophage colony-stimulating factor (GM-CSF) therapy has a significant effect on the reversal of the occurrence of HAI, organ dysfunction recovery, and levels of HLA-DR and TNF-α, without mortality benefits in IPMOF [7]. There is a paucity of literature on the effectiveness of filgrastim (G-CSF) in sepsis-associated immunosuppression in Indian children. The study cohort has immunocompetent children having evidence of infection/sepsis with MOFS persisting beyond day three. This exploratory trial evaluates the effect of filgrastim (G-CSF) in sepsis-induced immunosuppression in immunocompetent children. We used clinical criteria of different phenotypes of sepsis-induced MOFS to represent real-world pediatric intensive care unit (PICU) settings where facilities for laboratory estimation of biomarkers are not available. However, biomarkers were estimated at the end of the study in stored samples, and subgroup analyses of biomarker-based IPMOF were discussed. 

## Materials and methods

This exploratory trial was conducted from March 2023 to June 2024 in Medical College Hospital, situated in northern India, in children with MOFS beyond day three. The study was approved by the Institute Ethics Committee (No. Dean/2022/EC/3642) and prospectively registered with the Clinical Trials Registry of India (CTRI/2023/03/050862). The children aged 1 month to 18 years with systemic inflammatory response syndrome with suspected or proven sepsis were monitored for the development of organ failures. The Goldstein criteria [8] of organ failure score were used to define MOFS, and two or more organ failures persisting for three days were the inclusion criteria; the children were randomized. We excluded TAMOF, SMOF, children with serum ferritin > 500 mg/dl, hematological diseases (neoplasms, leukemia), and transplant patients. The definition of phenotypes of MOFS (IPMOF, TAMOF, SMOF) was based on clinical criteria proposed by Carcillo et al. [4] at the time of randomization, representative of real-world PICU settings. IPMOF was defined as two or more organ failures persisting for three days. TAMOF was defined as new thrombocytopenia < 100 K, or if the baseline platelet count is < 100 K, then a 50% decrease from baseline, elevated LDH > 250 u/L, creatinine > 1 mg/dL, and oliguria. SAMOF was defined as PaO2/FiO2 < 300 with the need for mechanical ventilation, followed days later by new-onset hepatic dysfunction, ALT > 100 u/L + bilirubin > 1 mg/dL. The estimation of biomarkers: TNF-α, ADAMTS13, and soluble FasL in stored blood samples was done at the end of the study. Diagnosis of ventilator-associated pneumonia (VAP) and catheter-associated urinary tract infection (CAUTI) was based on criteria given by the Centers for Disease Control and Prevention (CDC) [9,10]. A randomization sequence was generated by an independent party, and the children were randomized into two groups using random permuted blocks of 4, 6, and 8, and allocation of children was done by using numbered opaque and sealed envelopes. Data entry and analysis were done by an independent party not involved in recruitment, randomization, allocation, and treatment. A detailed history and examination were recorded, including pediatric index of mortality (PIM-3), pediatric sequential organ failure assessment score (pSOFA), demographic profile, anthropometry, laboratory test results, cultures, diagnosis, treatment, health care-associated infection (HAI), survivor and non-survivor, and subsequent course of hospitalizations. The primary outcome was 28-day mortality (alive at 28 days) between the groups, and the secondary outcomes were the difference in TNF-α & pSOFA at randomization and the difference in TNF-α, pSOFA, and HAI at day seven after randomization. The intervention group received standard management plus filgrastim (G-CSF) at 4 mcg/kg/day subcutaneously for seven days, unless discontinued earlier due to mortality, leave against medical advice (LAMA), or elevated total leukocyte count (TLC > 30,000 cells/mm³) [5]. The control group received standard management without filgrastim (G-CSF). The rationale for seven days of use of filgrastim (G-CSF) was based on the evidence that persistently low levels of TNF-α at the end of the first week are an independent risk factor for adverse outcomes (mortality, nosocomial infection, and persistent organ dysfunction) [5,6,11,12]. The cutoff of TLC >30000 cells/mm³ was based on a study done by Hall et al. [5]. Baseline blood samples were collected at randomization, i.e., day 0, to estimate biomarkers (TNF-α, ADAMTS13, FasL), and a second blood sample was collected on day seven after randomization for TNF-α estimation. Blood samples were collected aseptically in plain and heparin vials, and separated supernatant and serum samples were stored at -80°C at the departmental research lab. Lipopolysaccharide-induced tumor necrosis factor-alpha (LPS-induced TNF-ɑ), ADAMTS13, and FasL were measured by chemiluminescence using the Immulite automated chemiluminometer with the human ELISA kit from Fine Test as per the manufacturer's guidelines. A total of 78 samples for LPS-induced TNF-ɑ, ADAMTS13, and FasL (39 each from intervention and control) were processed at day 0, and a separate 45 samples for LPS-induced TNF-ɑ (25 from intervention and 20 from control) were processed at day seven.

Processing of the sample

Blood samples were collected aseptically in plain and heparin vials. From heparinized vials within 1 h of collection, a 50 microliter sample was mixed with 500 microliters of phenol-extracted LPS from *Salmonella abortus equi* (500 pg/mL), which stimulates TNF-alpha, and incubated at 37°C for 4 hours. After centrifugation for 5 min at 1000 rpm, the supernatant was then separated and stored at -80°C. The samples from the plain vial were allowed to clot at room temperature for 30 min, and later serum was separated by centrifugation at 1000 rpm for 10 minutes. The separated supernatant and serum samples were stored at -80°C in the departmental research lab. The stored samples were thawed and then analyzed for levels of biomarkers (TNF-α, ADAMTS13, FasL). A reagent standard working solution was prepared as per the manufacturer's instructions, and 100 µl of the samples were loaded into the appropriate wells. The sealed plates were incubated at 37 degrees for 90 minutes. The wells were then washed with a pre-prepared wash solution. In the next step, 100 µl of biotinylated antibody working solution was added to each well, and sealed plates were incubated for 60 minutes at 37 degrees. The wells were again washed. 100 µl of Streptavidin HRP working solution was added to each well and incubated at 37 degrees for 30 minutes. After one more cycle of washing, 90 µl of TMB substrate solution was added to each well and incubated at 37 degrees in the dark for 10-20 minutes. The liquid turns blue with the addition of TMB solution. Then, 50 µl of stop reagent is added to each well. The liquid will turn yellow upon the addition of the stop reagent. The microplate reader was run, and measurement was conducted at 450 nm in an Immulite automated chemiluminometer. Biomarker (TNF-α, ADAMTS13, FasL) values were charted on a curve with optical density, and final values were derived after multiplication by the dilution factor. Whole-blood ex vivo LPS-induced TNF-α response < 200 pg/mL, ADAMTS13 activity < 57% of control, and FasL > 200 pg/ml were considered abnormal.

Sample size and statistical analysis

To decrease the mortality by 50% in the intervention group, with 80% power, a 5% level of significance, and accounting for 10% attrition, a sample size of 42 patients per group was required. Data were analyzed using IBM Corp. Released 2021. IBM SPSS Statistics for Windows, Version 27. Armonk, NY: IBM Corp. Categorical variables were described as frequencies and percentages, while numerical variables were presented as medians and interquartile ranges (IQR). The Mann-Whitney U test was used for continuous variables, and Pearson's chi-square or Fisher's exact test for categorical variables. A p-value of <0.05 was considered statistically significant. An intention-to-treat analysis was done to find out the mortality benefits of filgrastim (G-CSF). The primary outcome was mortality, and we assumed a 10% attrition rate; only 3.8% of children went LAMA. They were treated as mortality, so there was no/minimal missing data.

## Results

**Figure 1:**
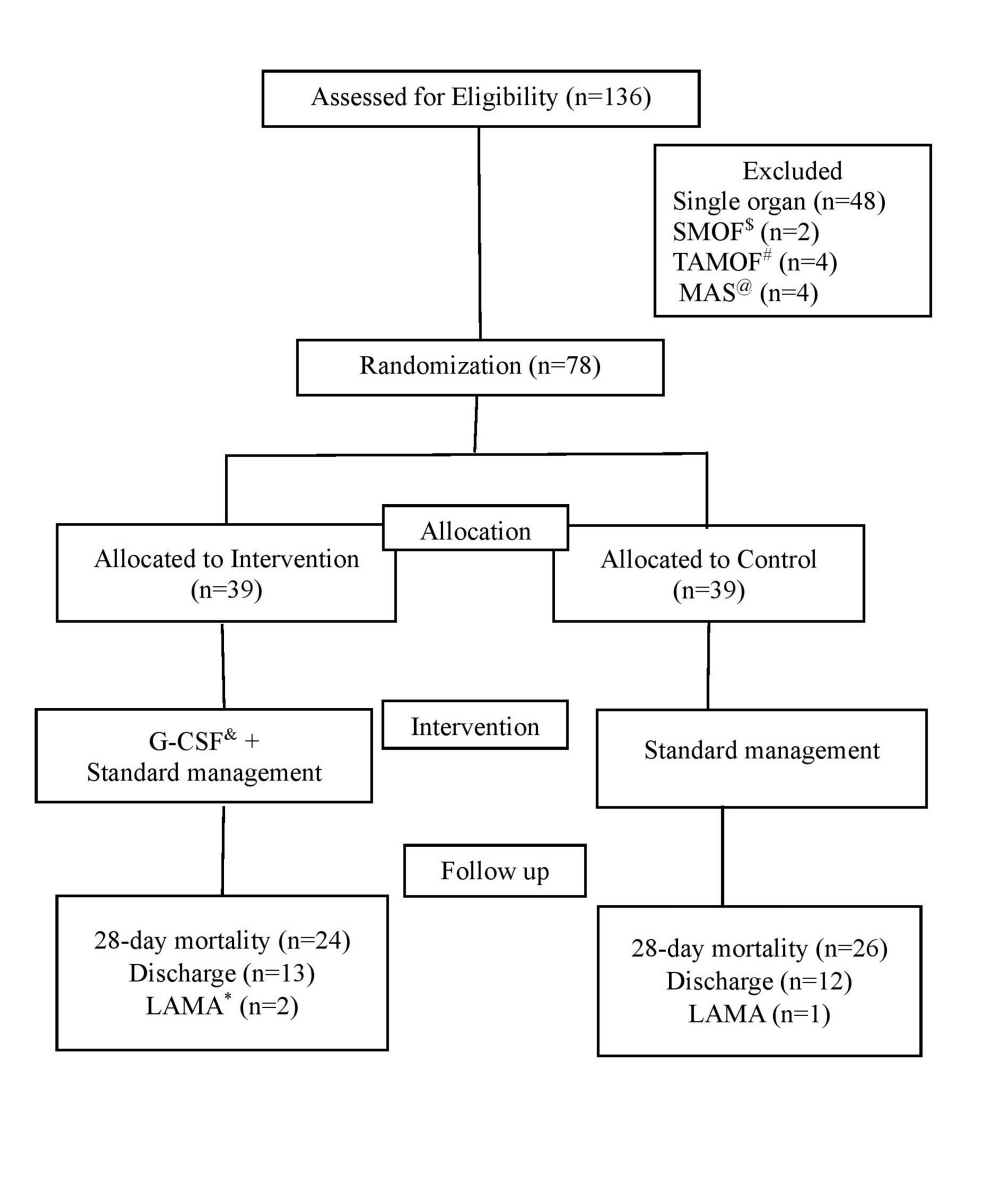
Consort diagram of the flow of participants in the study $ SMOF: Sequential multi-organ failure, # TAMOF: Thrombocytopenia-associated multi-organ failure, @ MAS: Macrophage-activation syndrome, & G-CSF: Granulocyte-Colony Stimulating Factor, *LAMA: left against medical advice

One hundred thirty-six children were assessed for eligibility; out of these, 78 children were randomized into intervention (39) and control groups (39), and 58 children were excluded (Figure 1). Of the 78 children, 25 (32%), 50 (64.1%), and 3 (3.8%) were discharged, died, and LAMA, respectively. The baseline characteristics of the study population, like age, anthropometry, blood biochemistry, shock, pSOFA, PIM-3, organ dysfunction, and standard treatment, including invasive mechanical ventilation (IMV), non-invasive mechanical ventilation (NIV), inotropes, blood component therapy, and dialysis, are shown in Table 1. The two groups were similar except for a higher TLC (p=0.02) and male preponderance (p=0.03) in the control group. However, there was no significant difference observed between TLC (p=0.25) and male preponderance (p=0.63) in survivors vs. non-survivor children. Of 78 children with MOFS, 47 (60.2%), 25 (32%), 3 (3.8%), and 3 (3.8%) were IPMOF (26-intervention and 21-control), unclassified MOFS (10-intervention and 15-control), TAMOF (3-control), and SMOF (3-intervention), respectively. The primary outcome (mortality) and secondary outcome (TNF-α, pSOFA, and HAI) variables are shown in Table 2. The mortality was comparable in both groups at day 28 (95% CI, 0.71-1.31, p=0.81), and there was a similar proportion of mortality in IPMOF (95% CI, 0.60-1.42, p=0.71) at 28 days. The median (IQR) value of TNF-α levels and pSOFA scores in children with MOFS beyond day three and children with IPMOF were comparable at day zero (81.6 [6.9-237.2] vs. 99.9 [16.2-404.2], p=0.41) and (19.4 [6.9-118] vs. 44.4 [16.5-101]), p=0.32) and day seven (84.2 [25-354.7] vs. 44.9 [8.7-207.9], p=0.29), respectively. The median (IQR) pSOFA scores at day zero (7 [6-9] vs. 8 [6-8], p=0.56) and day seven (9 [.5-10.5] vs. 7 [4.5-9.5], p=0.22) were also comparable. A total of 65 non-bronchoscopic bronchoalveolar lavage (BAL) samples and 50 urine samples were screened for HAI, and the frequency of HAI was 52/115 (45.2%). and it was equally observed in both groups (31/62 vs. 21/53, p=0.35). BAL culture positivity was 42/65 (64.6%), and CAUTI was 10/50 (25%). *Acinetobacter baumanii*, 29/65 (44.6%) among ventilator-associated pneumonia and *Candida*, and 6/50 (1.5%) among CAUTI, were prevalent organisms that grew in culture. Out of 39 children, 2 (5.1%), 17 (43.5%), 12 (30.7%), 6 (15.3%), and 2 (5.1%) children received 1, 2, 3, 4, and 5 doses of filgrastim (G-CSF), respectively. The reasons for stopping filgrastim (G-CSF) were elevated total leukocyte count in 23 (58.9%), mortality in 14 (35.8%), and 2 (5.1%) due to LAMA. The median (IQR) dose and duration of filgrastim (G-CSF) in intervention groups were 2 (2-3) and 3 (2-3), respectively. There was a trend of increased levels of TNF-α in intervention group children with MOFS beyond day three at day seven (84.2 [25-354.7] vs. 44.9 [8.7-207.9], p=0.29) as compared to control children with MOFS beyond day three. Similarly, there was also a trend of increased levels of TNF-α in intervention group children with IPMOF at day seven (56 [32-118] vs. 35 [33-86], p=0.62) as compared to control children with IPMOF. Within the intervention group, the level of TNF-α in children with MOFS beyond day three at day zero and day seven was 81.6 (6.9-237.2) vs. 84.2 (25-354.7); p=0.98, whereas the level of TNF-α in children with IPMOF at day zero and day seven was 19.4 (6.9, 118) vs. 56 (32, 118); p=0.77 (Table 2). Within the control group, the level of TNF-α in children with MOFS beyond day three at day zero and day seven was (99.9 [16.2-404.2] vs. 44.9 [8.7-207.9]; p=0.22), whereas the level of TNF-α in children with IPMOF at day zero and day seven was (44.4 [16.5,101] vs. 35 [33,86]; p=0.97) (Table 2).

**Table 1:** Baseline characteristics of the study population *MOFS: Multi-organ failure syndrome, ! IPMOF: Immunoparalysis-associated multi-organ failure syndrome, & pSOFA: Pediatric sequential organ failure assessment, + IMV: Invasive mechanical ventilation, #: Mann-Whitney U test, $: Pearson chi-square test, @: Fischer exact test.

Baseline characteristics	Intervention, (n=39) median(IQR), n(%)	Control, (n=39) median(IQR), n(%)	p-value
Age (months)	42 (12-156)	72 (36-132)	Z=0.71, 0.48^#^
Male	18 (46.2)	27 (69.2)	X^2^=4.26, 0.039^$^
Body Mass Index (kg/m^2^)	13.7 (12.2-15.5)	14 (12.1-15.2)	Z=0.12, 0.90^#^
pSOFA^&^	7 (6-9)	8 (6-8)	Z=0.58, 0.56^#^
Pediatric Index of Mortality-3	18.1 (7.6-34.4)	18.3 (6.5-50.6)	0.84^#^
Hemoglobin, g/dl mean (SD)	10.4 (1.85)	10.1 (2.2)	t=0.56, 0.57^#^
Leucocyte count	14100 (11400-16270)	17560 (13900-22100)	Z=3.03, 0.02^#^
Platelets	190000 (122000-299000)	220000 (141000-320000)	Z=0.66, 0.50^#^
C reactive protein, mg/l	22 (10.9-62.4)	32.3 (12.1-112)	^Z=1.32, 0.19#^
Urea, mg/dl	45 (24-92.7)	37 (27-68)	Z=0.005, 1^#^
Creatinine, mg/dl	0.74(0.4-1.3)	0.7(0.4-1.6)	Z=0.54, 0.58^#^
Blood culture, n (%)	9 (23.1)	5 (12.8)	0.38^@^
Organ dysfunction, n (%)			
Cardiovascular System	39 (100)	37 (94.8)	0.49^@^
Central Nervous System	22 (56.4)	30 (76.9)	X^2^=3.69, 0.06^$^
Renal	18 (46.2)	18 (46.1)	X^2^=0.00, 1.00^$^
Hepatic	18 (46.2)	13 (33.3)	X^2^=1.34, 0.25^$^
Respiratory	15 (38.5)	16 (41)	X^2^=0.05, 0.81^$^
Hematological	5 (10.3)	4 (10.2)	1.0^@^
Shock at Presentation	31 (79.5)	28 (71.8)	X^2^=0.63, 0.42^$^
Standard Treatment, n (%)			
Mechanical Ventilation	38 (97.4)	36 (92.3)	0.30^@^
Non-Invasive Ventilation	24 (61.5)	20 (51.5)	0.36^@^
Inotropes	39 (100)	36 (92.3)	0.07^@^
Blood Transfusion	21(53.8)	23 (58.9)	X^2^=0.21, 0.64^$^
Fresh Frozen Plasma	6 (15.4)	10 (25.6)	X^2^=1.26, 0.26^$^
Dialysis	2 (5.1)	7 (17.9)	0.07^@^
IMV^+^ Duration	7 (5-7)	6 (5-7.2)	0.14^#^
Duration of Stay	11 (7-14)	7 (6-9.5)	0.005^#^
Inflammatory phenotypes of MOFS (Clinical and/or Biomarker based)			
Children with MOFS^*^ beyond day-3, n=78	39(100)	39 (100)	
Children with IPMOF^!^, n=47	26 (66.6)	21(53.8)	0.34^@^
Children with TAMOF^!^, n=3	0	03(7.6)	
Children with SMOF^!^, n=3	03 (7.6)	0	
Unclassified MOFS, n=25	10 (25.6)	15 (38.4)	0.33^@^

**Table 2:** Primary and secondary outcome variables of study cohort % MOFS: Multi-organ failure syndrome, *IPMOF: Immunoparalysis-associated multi-organ failure, $ TNF-α: Tumor necrosis factor-α, pSOFA: Pediatric sequential organ failure assessment score, &VAP: Ventilator-associated pneumonia, ! CAUTI: Catheter-associated urinary tract infection, #: Pearson chi-square test, @: Mann-Whitney U test, +: Fischer exact test.

Outcome Variables	N	Intervention, n (%) n=39	N	Control, n (%) n=39	p-value
Primary Outcome					
28-day mortality in Children with MOFS^%^ beyond day-3	39	26 (66.6)	39	27 (69.2)	X^2^=0.06, 0.81#
28-day mortality in Children with IPMOF^*^ (TNF-α<200 pg/ml)^$^	26	16 (61.5)	21	14 (66.6)	X^2^=0.13, 0.71#
Secondary Outcome, at Randomization (Day-0), median (IQR)					
TNF-α, (pg/ml)	39	81.6 (6.9-237.2)	39	99.9 (16.2-404.2)	0.41^@^
TNF-α< 200 pg/ml (IPMOF)	26	19.4 (6.9-118)	21	44.4 (16.5-101)	0.32^@^
pSOFA	39	8 (7-10)	39	9 (8-10)	0.24^@^
at Day-7, median (IQR)					
TNF-α, (pg/ml)	25	84.2 (25-354.7)	20	44.9 (8.7-207.9)	0.29^@^
TNF-α< 200 pg/ml (IPMOF)	17	56 (32-118)	11	35 (33-86)	0.62^@^
pSOFA	25	8(6-12)	20	9(8-11)	0.12^@^
Health Care Associated Infection (HAI), n (%)					
HAI	62	31 (50)	53	21 (39.6)	X^2^=1.24, 0.27^#^
VAP^&^	35	25 (71.4)	30	17 (56.6)	X^2^=1.54, 0.22^#^
CAUTI^!^	27	6 (22.2)	23	4 (17.3)	0.74^+^

## Discussion

The present study provides important insight into the safety and efficacy of filgrastim (G-CSF) in immunocompetent children with sepsis-induced MOFS beyond day three, which is underrepresented in the literature. Thirty-nine children, including 26 IPMOF and 3 SMOF children in the intervention group, received a 2 (2-3) median dose of filgrastim (G-CSF) for a 3 (2-3) median day duration of intervention. The intended seven-day filgrastim (G-CSF) dosing was rarely achieved, with a median of only two doses administered for a median of three days, and a 50% reduction in mortality underpowered the trial against finding a treatment effect and limits the interpretability of immunological and clinical outcomes. A more conservative effect size, e.g., 15-20% absolute risk reduction, would have provided a more realistic estimate of the required sample size, and exclusion of early death, i.e., within 48 hours, would have led to the intended seven-day exposure to filgrastim (G-CSF). We did not observe any life-threatening/significant sudden deterioration/anaphylaxis after use of filgrastim (G-CSF). The absence of severe adverse events is reassuring, though the trial was not powered to detect rare adverse events. A retrospective study of 109 pediatric sepsis patients found that those receiving immunomodulatory therapy, including filgrastim (G-CSF), required more frequent PICU admission and invasive ventilation [13]. In the present study, we observed equal requirements of invasive and non-invasive mechanical ventilation with nearly equal duration of invasive mechanical ventilation in both groups. This observation might be because of the equal distribution of classified phenotypes (26 intervention and 21 control) and unclassified phenotypes (10 intervention and 15 control) between the groups. The mortality rates were high and nearly identical across the groups and subgroups; these results are consistent with prior studies and meta-analyses conducted in adult populations [7,11,14]. The mortality results should be interpreted as inconclusive rather than confirmatory, given the small sample size and underdosing of filgrastim (G-CSF), which makes any mortality inferences statistically and pharmacologically underpowered. The lack of mortality reduction might be attributed to the severity of organ dysfunction, filgrastim (G-CSF) dose and administration timing, or a persistent sepsis-induced immunosuppression state due to the different case mix in the present cohort. Moreover, the timing of filgrastim (G-CSF) administration may be crucial. In adult studies, the delayed administration of filgrastim (G-CSF), often initiated after the onset of septic shock, was associated with a lack of benefit in terms of survival because it does not reverse immune dysfunction or halt disease progression [14]. In contrast to this, the use of filgrastim (G-CSF) in neonates with sepsis and neutropenia revealed mixed results in terms of mortality benefits. Chaudhuri et al. reported reduced mortality (10% in the intervention group vs. 35% in the control) in a randomized trial [15]. Similarly, Gathwala et al. demonstrated significantly lower mortality in preterm babies treated with rhG-CSF (3/20 vs. 7/20) [16]. However, Khan TH et al. [17] and Carr R et al. [18] found no mortality benefit in neonates. In contrast to this, the present study did not observe mortality benefits, likely due to the greater severity of organ dysfunction and the lower dose (4 mcg/kg/day S/C for seven days) compared to the higher doses (10 mcg/kg/day IV) used in these trials.

The prior studies have shown that filgrastim (G-CSF) or GM-CSF therapy significantly increased the reversal rate of the infection by increasing expression of mHLA-DR and levels of LPS-induced TNF-ɑ [11,19]. There was a trend of increased levels of TNF-α in intervention groups of children with MOFS beyond day three at day seven (84.2 [25-354.7] vs. 44.9 [8.7-207.9]; p=0.29) as compared to control children with MOFS beyond day three. Similar trends were observed in children with IPMOF at day seven (56 [32-118] vs. 35 [33-86]; p=0.62) as compared to control children with IPMOF. The lack of a significant increase in TNF-α levels suggests no immunostimulatory effect of filgrastim (G-CSF) at the administered dose (two median doses) and duration (three median days). The timing of administration of filgrastim (G-CSF) may have an effect on levels of LPS-induced TNF-ɑ. However, the case mix of the present cohort had an overlap of inflammatory phenotypes, and nearly one-third of children had unclassified inflammatory phenotypes of MOFS, which could affect the efficacy of filgrastim (G-CSF), as pathobiological mechanisms are different [3]. In contrast to this, Hall et al. demonstrated increased levels of mHLA-DR and LPS-induced TNF-ɑ by GM-CSF in immunocompromised and immunocompetent children who developed MOFS. The findings of the GRID randomized controlled trial were consistent with the present study; the authors evaluated the efficacy of GM-CSF in preventing ICU-acquired infection and 28-day mortality in septic adults and found that there was no significant difference observed between groups regarding ICU-acquired infection (11% vs. 11%, p=1.00) and 28-day mortality (24% vs. 27%, p=0.90) [11]. While TNF-ɑ is appropriate for identifying immunoparalysis, the absence of monocyte HLA-DR measurement, a gold standard marker of innate immune suppression, limits immune profiling, and the clinical relevance of a modest increase in LPS-induced TNF-ɑ levels without corresponding changes in HAI or pSOFA is unclear. The absence of a significant reduction in pSOFA scores indicates that filgrastim (G-CSF) may not have an impact on reversing organ failure scores once it has developed, suggesting that its immunomodulatory effects are limited in the context of advanced sepsis and persistent low levels of TNF-α at day seven, as demonstrated in the present study and prior studies [5,11].

The present trial explores the targeted therapy for an important contributor to morbidity and mortality in children with sepsis-induced MOFS beyond day three. The facility for estimation of biomarkers (TNF-ɑ, ADAMTS13, FasL) is not widely available in resource-limited settings; therefore, we decided to recruit and randomize children based on clinical criteria of inflammatory phenotypes of sepsis-induced MOFS proposed by Carcillo et al. as representative of real-world PICU settings. The confirmation of IPMOF, TAMOF, and SMOF by estimation of biomarkers, TNF-α, ADAMTS13, and FasL in the stored blood samples at the end of the study. We demonstrated 47 (60.2%) children with IPMOF and 3 (3.8%) children in each phenotype, TAMOF and SMOF, with significant contribution in mortality by IPMOF, 30/47 (63.8%). The longitudinal TNF-α trends (day 0 to day 7 within each group) suggested modest increases in levels of TNF-α in intervention group children with IPMOF at day seven (19.4 [6.9,118] vs. 56 [32,118]; p=0.77) and modest decreases in levels of TNF-α in control group children with IPMOF at day seven (44.4 [16.5,101] vs. 35 [33,86]; p=0.97). Filgrastim (G-CSF) prevented further suppression of TNF-α at an administration of two doses for three days. The underdosing and limited exposure of filgrastim (G-CSF) underpowered the trial to detect the cumulative dose and duration of filgrastim (G-CSF), which will be required to recover organ dysfunction, prevent HAI, and ultimately benefit mortality. The study addresses an important and underexplored clinical question: whether filgrastim (G-CSF) can modulate immune function and improve outcomes in immunocompetent children with sepsis-induced MOFS, particularly IPMOF. Key strengths include the prospective randomized design, incorporation of inflammatory phenotyping, use of objective biomarkers (TNF-ɑ, ADAMTS13, FasL), and focus on a high-risk pediatric population in a resource-limited setting. The limitations of the present study include underpowering due to an overly optimistic effect size assumption, lack of blinding, single center, short-term follow-up, underdosing, and limited duration of administration of filgrastim (G-CSF), and limited immune profiling, with no mHLA-DR data and minimal functional immune assessment and phenotype heterogeneity. However, findings of the present trial provide a platform for future studies with a conservative effect size (15-20% absolute risk reduction), which provide a realistic estimate of the required sample size, recruitment, and randomization based on clinical and biomarker levels to avoid phenotype heterogenicity, functional immune assessment (mHLA-DR expression), and consideration of factors responsible for medication discontinuation (early deaths, LAMA, and TLC triggers) to better understand the dose and duration of administration of filgrastim (G-CSF) in IPMOF.

## Conclusions

The immunocompetent children with sepsis-induced MOFS, particularly IPMOF, which is underrepresented in the literature, make a significant contribution to morbidity and mortality in sepsis-induced MOFS. Filgrastim (G-CSF) use in immunocompetent children with sepsis is safe, and a median dose of 2 G-CSF for three median days has poor efficacy in the reduction of mortality and did not show significant change in frequency of HAI, TNF-α levels, and pSOFA scores. Future studies with a conservative effect size (15-20% absolute risk reduction), recruitment and randomization based on clinical and biomarker levels, functional immune assessment by mHLA-DR expression, and consideration of factors responsible for medication discontinuation would be required to better understand the dose and duration of administration of filgrastim (G-CSF) for restoration of innate immune suppression in immunocompetent children with sepsis.
